# Identification, characterization and classification of prokaryotic nucleoid‐associated proteins

**DOI:** 10.1111/mmi.15298

**Published:** 2024-07-22

**Authors:** Samuel Schwab, Remus T. Dame

**Affiliations:** ^1^ Leiden Institute of Chemistry Leiden University Leiden The Netherlands; ^2^ Centre for Microbial Cell Biology Leiden University Leiden The Netherlands; ^3^ Centre for Interdisciplinary Genome Research Leiden University Leiden The Netherlands

**Keywords:** AlphaFold, histone, nucleoid, nucleoid‐associated proteins

## Abstract

Common throughout life is the need to compact and organize the genome. Possible mechanisms involved in this process include supercoiling, phase separation, charge neutralization, macromolecular crowding, and nucleoid‐associated proteins (NAPs). NAPs are special in that they can organize the genome at multiple length scales, and thus are often considered as the architects of the genome. NAPs shape the genome by either bending DNA, wrapping DNA, bridging DNA, or forming nucleoprotein filaments on the DNA. In this mini‐review, we discuss recent advancements of unique NAPs with differing architectural properties across the tree of life, including NAPs from bacteria, archaea, and viruses. To help the characterization of NAPs from the ever‐increasing number of metagenomes, we recommend a set of cheap and simple in vitro biochemical assays that give unambiguous insights into the architectural properties of NAPs. Finally, we highlight and showcase the usefulness of AlphaFold in the characterization of novel NAPs.

## Graphical abstract

Nucleoid‐associated proteins (NAPs) structure DNA by either bending, wrapping, bridging, or forming protein filaments on the DNA. We review newly identified NAPs and provide a list of simple biochemical assays to study the DNA‐structuring properties of novel NAPs.

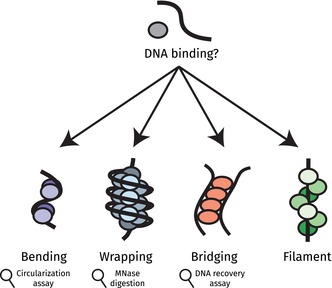

## 1 INTRODUCTION

The DNA genome is both the most conserved molecule of life and the most diverse: all cells have it, yet it defines each cell uniquely. It encodes all information needed to function as an organism and for an organism to replicate itself. Although the order of the base pairs varies markedly between organisms, from a physical perspective, their genomes behave in the same way. As such, any problems that arise from its physical properties are shared between all life forms. One of the major problems faced by the genome is its most basic property: it takes up space.

Bacteria have to condense the volume of their genome 1000‐fold for it to fit inside their cell volume (Holmes & Cozzarelli, 2000). Despite several decades of research, how prokaryotes achieve this enormous level of compaction is still not well understood. Five possible mechanisms are often discussed as being involved in inducing compaction: supercoiling, phase separation, charge neutralization, macromolecular crowding, and nucleoid‐associated proteins (NAPs) (Joyeux, 2015). None of these mechanisms alone provide sufficient levels of compaction for the genome to fit inside the cell. In prokaryotes, multiple mechanisms likely synergize to organize the genome into a structure that we refer to as the nucleoid.

Most research focuses on how NAPs organize the nucleoid. NAPs are small proteins that bind DNA. From in vivo studies, we know that NAPs can affect transcription, DNA repair, replication, and recombination (Dame et al., 2020). From in vitro experiments, we know that NAPs can compact or decompact DNA, bend or wrap DNA, bridge DNA, and form protein filaments on the DNA (Luijsterburg et al., 2008). The dualistic role of NAPs in both DNA organization and basic cellular functions suggests that they are linked. Indeed, changes in the three‐dimensional DNA organization can affect transcription (Rashid et al., 2023).

The need to compact the genome into the nucleoid is universal across prokaryotes. It is then remarkable that across the tree of life, there is not one NAP universal to all life on earth, although structural maintenance of chromosomes (SMC) proteins or RNA polymerase are candidates depending on your definition of a NAP (van Hooff et al., 2024). In archaea, histones are the most common NAP. Histones are found in all eukaryotes and the vast majority of archaea. In eukaryotes, histones form nucleosomes: a cylindrical protein core around which ~147 bp of DNA is wrapped (Luger et al., 1997). Histones were long considered to be a eukaryotic invention. However, three decades ago two histones were discovered in the single‐celled archaeon *Methanothermus fervidus*: HMfA and HMfB (Sandman et al., 1990). HMfA and HMfB form nucleosome‐like structures, called hypernucleosomes, and are the ancestor histones from which the eukaryotic histones have evolved (Bailey et al., 2000; Henneman et al., 2021; Henneman & Dame, 2015; Henneman, Emmerik, et al., 2018; Mattiroli et al., 2017). HMfA and HMfB are ‘simple’ compared to the eukaryotic histones; they lack the long histone tails found in eukaryotic histones and the hypernucleosome structure does not require multiple different types of histones. Instead, hypernucleosome structures can be made from just HMfA proteins or just HMfB proteins. The simpler nature of HMfA and HMfB overlaps with the simpler lifestyle of archaea, in contrast to the complex lifestyle of eukaryotes. In bacteria, the histone‐like protein from *Escherichia coli* (*E. coli*) strain U93 (HU) is the most common NAP (Grove, 2011). HU is the NAP that ‘can do it all’. In vitro, it can bend DNA and form nucleoprotein filaments (Hodges‐Garcia et al., 1989; van Noort et al., 2004). In vivo, it is involved in transcription, DNA repair, recombination, and replication (Kamashev & Rouviere‐Yaniv, 2000; Karaboja & Wang, 2022; Prieto et al., 2012). Furthermore, evidence has been put forward that homologs of HU, such as the integration host factor (IHF) and histone‐like protein of *Thermoplasma acidophilum* (HTa), can bridge and wrap DNA (Hocher et al., 2019; Yoshua et al., 2021).

The variety of NAPs across prokaryotes is enormous. A single organism like *E. coli* already encodes 12 different NAPs. Furthermore, InterPro, a protein classification resource, contains more than 2000 unique bacterial DNA‐binding protein families (Blum et al., 2021). NAPs can be classified in three ways: based on protein structure, in vivo functions, or how they act in DNA organization. Structure classification might seem the best choice as protein structure gives rise to function. However, structural diversity between NAPs is enormous and thus it is unsuitable as a form of general NAP classification. In vivo function classification is a good alternative as proteins are used by cells for their in vivo functions. However, in vivo experiments are limited to model organisms. In contrast, the structural effects that NAPs have on DNA can readily be studied in vitro. Since DNA organization is thought to be tightly linked to in vivo function, classifying NAPs based on how they structure DNA is both practical and insightful. NAPs can be categorized into four different groups: those that bend DNA, wrap DNA, bridge DNA, or form nucleoprotein filaments (Luijsterburg et al., 2008).

In this mini‐review, instead of focusing on the well‐established NAPs in *E. coli*, we highlight the diversity of NAPs across the tree of life. We discuss recent findings on NAPs with differing DNA structuring properties in archaea, bacteria, and viruses. Furthermore, we discuss a new and interesting NAP from *E. coli* of which the architectural properties are still unknown. We use this new NAP as an example of how to make educated guesses about the DNA structuring properties of novel NAPs based on AlphaFold predictions. Finally, we shortly discuss cheap and easy assays that can readily categorize NAPs as bending, wrapping, or bridging proteins.

## 2 WHAT DEFINES A NAP?

Before discussing several specific NAPs, it is useful to define what exactly a NAP is. Commonly mentioned characteristics of NAPs include: they are small abundant proteins that structure DNA, have no sequence specificity and bind genome‐wide in intragenic and intergenic regions, and affect transcription and genome structure on a global level. In contrast, transcription factors (TFs) are not abundant, have strong sequence specificity, and bind intragenic regions. Although the NAP definition above is commonly recited, most proteins that we consider to be NAPs do not strictly adhere to these definitions. Instead, many NAPs share characteristics of TFs and vice versa. Regarding sequence specificity, IHF from *E. coli* is commonly regarded as an NAP even though it has a strong sequence preference (Yang & Nash, 1989). Other proteins, such as the leucine‐responsive regulatory protein (Lrp), can switch between sequence‐specific and aspecific DNA binding depending on the presence of a specific ligand (Peterson et al., 2007). On the other hand, TFs such as cAMP receptor protein (CRP) exhibit genome‐wide binding profiles (Grainger et al., 2005). While NAPs are viewed as architectural proteins, TFs also structure DNA upon binding. Examples of TFs that bend DNA are CRP, the nickel‐binding regulatory protein (NikR), and the locus for X‐ray sensitivity A protein (LexA) (Bracco et al., 1989; Schreiter et al., 2006; Zhang et al., 2010). TFs that can bridge DNA are the lac repressor (LacI) and the regulator of the L‐arabinose operon (AraC) (Lobell & Schleif, 1990; Rutkauskas et al., 2009).

One might argue that the main difference between NAPs and TFs is the scale of their architectural properties; NAPs globally structure the genome, while the effect of TFs is more local. We like to emphasize that the role of NAPs in global genome organization is not well understood. For example, histone‐like nucleoid structuring protein (H‐NS) from *E. coli* is universally referred to as a NAP and a global genome organizer. Indeed, ChIP‐seq shows that H‐NS binds globally on this genome (Grainger et al., 2006). However, the contribution of specific NAPs, such as H‐NS, to genome organization is difficult to study with current methods. Our lack of understanding of how proteins shape the genome on a global level makes it unsuitable as a definition for NAPs.

One could also split NAPs and TFs based on abundance; DNA‐binding proteins that are highly expressed and bind genome‐wide are more important in shaping the nucleoid and thus could be classified as NAPs. On the contrary, lowly expressed TFs that only bind at ~20 positions across the genome are less involved in shaping the nucleoid. This is indeed true if you would compare HU to LacI. However, cells express many different TFs, and thus as a group TFs are present everywhere on the nucleoid (Pérez‐Rueda & Collado‐Vides, 2000; Schmidt et al., 2016; Yilmaz & Schnetz, 2022). As many TFs deform DNA, they are involved in the structure of the nucleoid on a global level.

In the end, the distinction between NAPs and TFs is blurry. For a more extensive discussion on NAPs and TFs, see the opinion piece by (Dorman et al., 2020). In this review, we follow the definition as stated by the term NAP itself: any protein that is associated with the nucleoid.

## 3 BENDERS AND WRAPPERS

**FIGURE 1:**
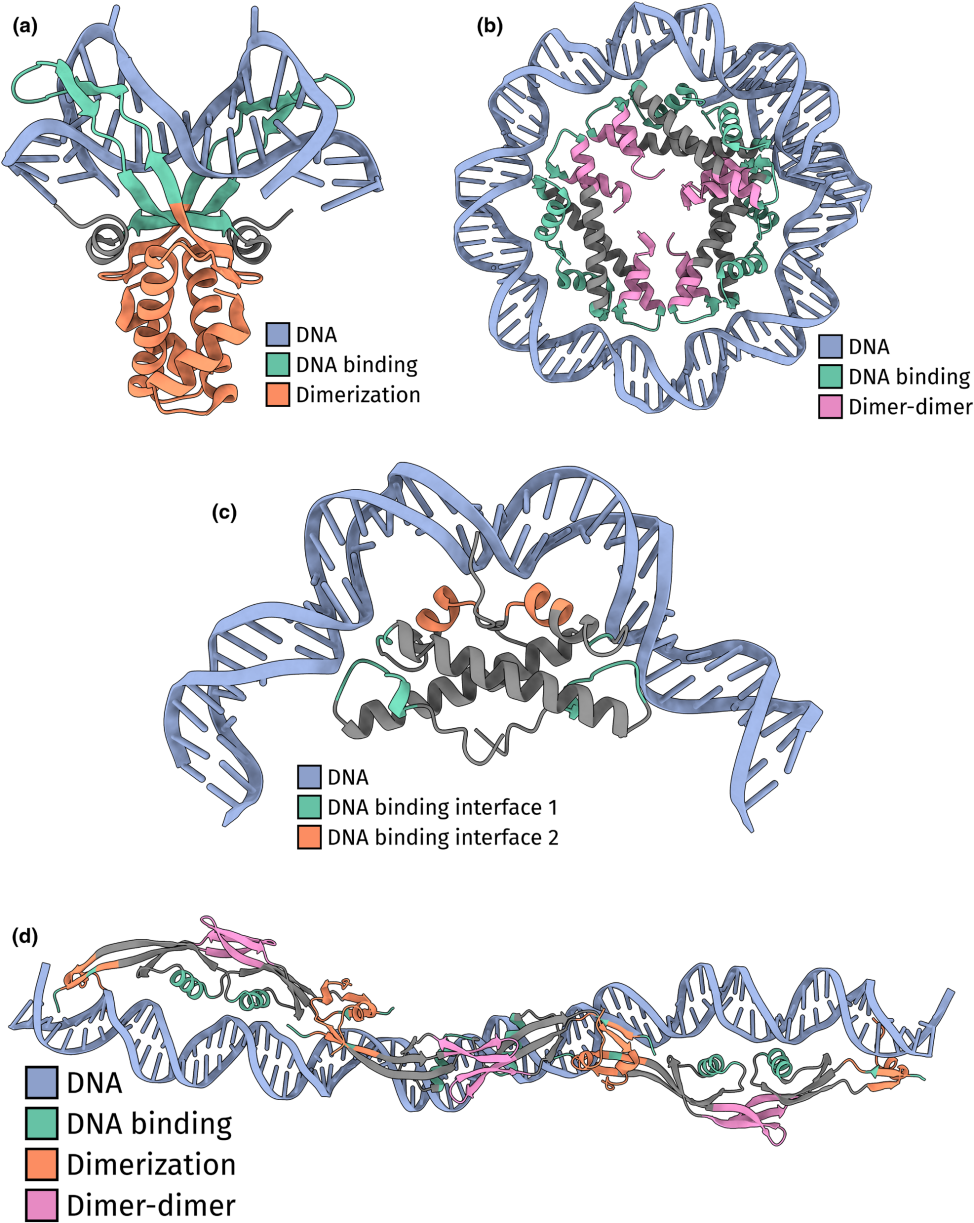
DNA bending and wrapping NAPs. (a) Model DNA bending NAP HU from *Nostoc* sp. (PDB: 1P51) (Swinger et al., 2003). The DNA, the DNA binding domain, and the dimerization domain are colored blue, green, and orange, respectively. (b) Model DNA wrapping NAP HMfB from *Methanothermus fervidus* (PDB: 5T5K) (Mattiroli et al., 2017). The DNA, the DNA binding domain, and the dimer‐dimer domain are colored blue, green, and pink, respectively. (c) The dimer structure of HBb/Bd0055 from *Bdellovibrio bacteriovorus* (PDB: 8CMP) (Hu et al., 2024). The DNA comes from a molecular dynamics simulation of HBB/Bd0055 with DNA (Hu et al., 2024). The DNA and the DNA binding interfaces 1 and 2 are colored blue, green, and orange, respectively. (d) The hexamer structure of protein p6 from *Bacillus subtillis* phage *ϕ*29 (PDB: 8PW4) (Alcorlo et al., 2024). The DNA comes from a docked structure of p6 with DNA generously provided by Martín Alcorlo and Juan A. Hermoso (Alcorlo et al., 2024). The DNA, the DNA binding domain, the dimerization domain, and the dimer‐dimer domain are colored blue, green, orange, and pink, respectively.

The majority of NAPs in prokaryotes are DNA benders. DNA benders induce small bends (<180°) into the DNA. They generally function as monomers or dimers. The defining member of DNA‐bending NAPs, and the first NAP to be identified in prokaryotes, is HU from *E. coli*. HU forms homo‐ and/or heterodimers in solution. The dimer structure forms a “saddle” shaped beta‐sheet structure that binds DNA nonspecifically and bends DNA up to 180° (Figure 1a) (Swinger et al., 2003). While the bending of DNA by HU compacts DNA, high concentrations of HU rigidify DNA (van Noort et al., 2004).

Wrappers differ from benders in that they induce stronger bends (>180°) and form larger multimers which are made up of individual bending units. Examples of DNA wrappers are the nucleosome and hypernucleosome histone (Luger et al., 1997; Mattiroli et al., 2017). Their multimer structures are built from individual histone dimers, with each histone dimer bending DNA, that together form a cylindrical protein core (Figure 1b) (Mattiroli et al., 2017). However, not all histones form wrapping multimers. Histone Bd0055/HBb from *Bdellovibrio bacteriovorus* functions as a dimer that bends DNA (Hu et al., 2024). As such, Bd0055/HBb is a simpler histone than the conventional histones as it does not form the larger nucleosome multimers. Besides the fact that this is the first DNA‐bending histone, it is also the first DNA‐binding histone protein identified in bacteria (Alva & Lupas, 2019; Hocher et al., 2023; Hu et al., 2024). Bd0055/HBb is highly expressed and essential to *Bdellovibrio bacteriovorus* (Hocher et al., 2023; Hu et al., 2024). Its structure contains the canonical histone fold although the C‐terminal helix of its histone fold is truncated (Figure 1c). The first co‐crystal structure of Bd0055/HBb suggested that it forms nucleoprotein filaments (Hocher et al., 2023). However, in a subsequent study, a second DNA‐binding interface was crystallized that suggests a different mode: DNA bending (Hu et al., 2024). In‐solution data and molecular dynamics simulations further support DNA bending as the form of DNA organization induced by Bd0055/HBb (Figure 1c) (Hu et al., 2024). While it is tempting to speculate that this simpler histone might be the ancestor histone of (hyper)nucleosome histones, it appears infrequently throughout the bacterial domain. Therefore, its origin could also be from a horizontal gene transfer event.

NAPs are not exclusively found in living organisms. Viruses also use proteins to structure their genomes (Bryson et al., 2022; Liu et al., 2021; Quemin et al., 2015). One of the earliest viral NAPs to be identified is protein p6 from *Bacillus subtillis* phage *ϕ*29 (Blanco et al., 1989). Protein p6 is highly abundant in infected *Bacillus subtilis* cells, with up to 700,000 copies per cell (Abril et al., 1997). Extensive biochemical research in the 1990s and the early 2000s discovered that p6 forms dimers and binds every 24 bps, compacts the viral genome by wrapping it around a multimeric core of p6, and restrains positive supercoiling (Prieto et al., 1988; Serrano et al., 1990, 1993). Besides its architectural role, p6 plays a crucial role in the initiation of replication and transcription. However, no structural data of p6 existed with and without DNA until recently. Alcorlo et al. (2024) show that p6 indeed forms a superhelical protein multimer, combining decades of research into one comprehensive model (Figure 1d). The authors succeeded in crystallizing apo and DNA‐bound p6, by truncating the disordered tail, as identified by AlphaFold, of protein p6. Protein p6 has a unique fold, consisting of four beta sheets, two alpha helices, and a C‐terminal disordered tail. It multimerizes “head to tail” forming a long chain of p6 dimers around which DNA is wrapped. Although “wrapping” is often mentioned as the mode of DNA compaction by p6, we like to note that the structural model of the p6‐DNA complex is more like a nucleoprotein filament, in some ways similar to filaments formed by H‐NS. It is then surprising that p6 compacts the viral genome since H‐NS is known to make DNA less compact when it forms nucleoprotein filaments without bridging (van der Valk, Vreede, et al., 2017). The data that support DNA compaction by protein p6 are electron microscopy images of fixed and dehydrated protein p6‐DNA complexes, and as such do not represent how protein p6 behaves in water (Gutiérrez et al., 1994; Serrano et al., 1993). Preferably, the architectural properties, specifically the ability to compact DNA, of p6 would be investigated with an in‐solution method like tethered particle motion (Henneman, Heinsman, et al., 2018; van der Valk, Laurens, & Dame, 2017).

## 4 BRIDGERS

Bridgers are special among NAPs in that they facilitate long‐range (distance ≫100 bp) DNA interactions. Bridgers generally form multimers. In these multimer structures, the DNA‐binding domains do not form one large binding domain but instead act independently from each other. Accordingly, each DNA binding domain binds a separate DNA duplex.

**FIGURE 2:**
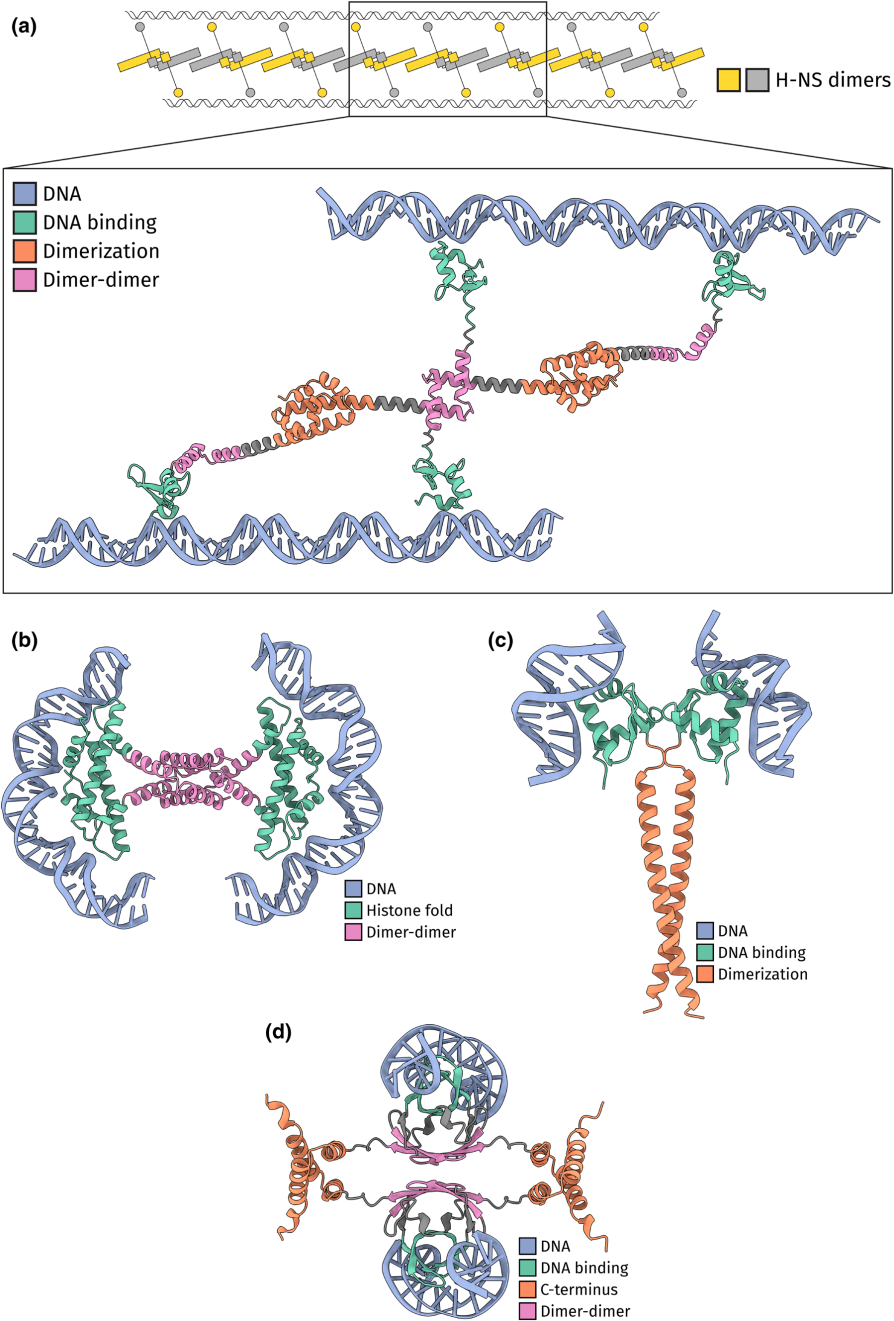
DNA bridging NAPs. (a) The DNA‐bridging filament formed by model DNA bridging NAP H‐NS from *Escherichia coli*. The zoom‐in shows the homotetramer of H‐NS as predicted by AlphaFold2. The DNA, the DNA binding domain, the dimerization domain, and the dimer‐dimer domain are colored blue, green, orange, and pink, respectively. The H‐NS dimers that make up the filament are colored gray and yellow. Placement of the DNA is based on H‐NS‐DNA models (Arold et al., 2010; Boudreau et al., 2018; Qin et al., 2019; van der Valk, Vreede, et al., 2017). (b) The homotetramer of MJ1647 from *Methanocaldococcus jannaschii* as predicted by AlphaFold2 (Ofer et al., 2023). The DNA, the DNA binding dimeric histone fold domain, and the C‐terminal dimer‐dimer domain are colored blue, green, and pink, respectively. The DNA was placed by superimposing the DNA‐binding histone domain from the DNA co‐crystal structure of HMfB from *Methanothermus fervidus* (PDB: 5T5K) (Mattiroli et al., 2017). (c) The homodimer of Sul12a from *Sulfolobus acidocaldarius* as predicted by AlphaFold2. The DNA, the DNA binding domain, and the dimerization domain are colored blue, green, and orange, respectively. The DNA was placed by superimposing the DNA‐binding domain of the DNA co‐crystal structure of IdeR from *Saccharopolyspora erythraea* (PDB: 7B1Y) (Marcos‐Torres et al., 2021). (d) The homotetramer of SymE from *Escherichia coli* as predicted by AlphaFold2. The (disordered) N‐terminal tail of SymE is hidden to reduce visual clutter. The DNA, the DNA binding domain, the dimerization domain, and the dimer‐dimer domain are colored blue, green, orange, and pink, respectively. The DNA was placed by superimposing the DNA‐binding domain of the NMR structure of MazE bound to DNA from *Escherichia coli* (PDB: 2MRU) (Zorzini et al., 2015).

Many bridging NAPs have been identified and extensively studied in bacteria (Erkelens et al., 2022; Qin et al., 2019). The defining member among bridgers is H‐NS from *E. coli*. H‐NS silences horizontally acquired genes (Lucchini et al., 2006). Important to its role as a silencer, it acts in genome organization multimerizing on DNA either forming nucleofilaments or DNA bridges (Dame et al., 2000; van der Valk, Vreede, et al., 2017). In its bridging mode, each monomer in the multimer can potentially bind a separate DNA duplex (Arold et al., 2010; Boudreau et al., 2018; Qin et al., 2019, 2020; van der Valk, Vreede, et al., 2017) (Figures 2a and S1). As such, H‐NS can bring multiple distant DNA sites close together in space. The multimers are formed through the dimerization and dimer‐dimer interfaces on the N‐terminus of H‐NS. The C‐terminus contains the DNA‐binding domain. In between the N‐ and C‐termini, H‐NS has a linker region which is important in regulating the mode of action, either bridging or filament formation, of H‐NS (Qin et al., 2020).

While many bridging NAPs have been identified in bacteria, we only know of a handful in archaea. Recently, the histone protein MJ1647 from *Methanocaldococcus jannaschii* was found to act as a DNA bridger (Ofer et al., 2023). This is surprising as all other known histones in archaea form a nucleosome‐like structure called the hypernucleosome. Hypernucleosomes do not bridge DNA but wrap DNA in a manner identical to the eukaryotic nucleosome (Henneman & Dame, 2015; Mattiroli et al., 2017). MJ1647 contains a canonical histone fold, with the addition of a C‐terminal extension in the form of two additional alpha helices (Figures 2b and S2). Among (hyper)nucleosome histones, the histone fold itself acts both as the DNA binding domain and the multimerization domain. While canonical histones form dimers in solution, MJ1647 forms a dimer of dimers. This tetramer is formed through the interaction of MJ1647's C‐terminal domains. As a result, the two histone fold dimers within the tetramer are connected through their C‐terminal extensions. As the histone dimer folds do not directly interact with each other, they act independently from each other and thus can bind separate DNA duplexes (Figure 2b). In contrast, canonical histone dimers multimerize through their histone folds and thus create one large DNA‐binding domain (Figure 1b). Removing the C‐terminal domain from MJ1647 removes its ability to form tetramers and to bridge DNA, highlighting that its unique multimerization is required for DNA bridging.

The best‐studied DNA bridging NAP in archaea is Alba. Alba is encoded in most archaeal genomes, although research primarily focuses on Alba from *Sulfolobus acidocaldarius* as it is one of the few archaeal model organisms. For a recent review on Alba, see Goyal et al. (2016). In the closely related species *Sulfolobus islandicus*, a new bridging NAP is found: the archaeal DNA condensing protein 1 (aDCP1, SiRe2004) (Zhang et al., 2023). All Sulfolobus species encode a copy of aDCP1, including *Sulfolobus acidocaldarius* (Sul12a, Saci1012) (Lemmens et al., 2022). Based on ChIP‐seq data, both aDCP1 and Sul12a bind evenly across the chromosome. Interestingly, aDCP1 induces phase separation in vitro in the presence of DNA. Phase separation is used by eukaryotes to organize their genomes (Strom et al., 2017). While phase separation is mentioned as a possible mechanism of genome compaction in prokaryotes, little evidence exists of NAPs that induce liquid–liquid phase separation (Feric & Misteli, 2021). Specifically, the DNA‐binding protein from starved cells (Dps), a NAP that co‐crystallizes with DNA to protect the genome, HU, and the host factor for bacteriophage Q*β* RNA (Hfq), a NAP that also has RNA chaperone functions, are mentioned as possible candidates that induce phase separation. For Dps and HU, in vitro data shows that they can form liquid droplet condensates with DNA, while Hfq forms condensates with RNA (Gupta et al., 2023). However, whether they compact the genome in vivo by liquid–liquid phase separation remains unknown. Similarly, for aDCP1 we do not know if it induces phase separation in vivo.

The structure of aDCP1 or Sul12a has not been solved experimentally, although AlphaFold predictions can give insights into its structure and function (Evans et al., 2021; Jumper et al., 2021). Based on its AlphaFold prediction, aDCP1/Sul12a contains an N‐terminal winged‐helix domain, which is likely the DNA binding domain, and a C‐terminal alpha helix (Figures 2c and S3). Interestingly, the overall structure of aDCP1/Sul12a is highly similar to the *Saccharolobus solfataricus* NAPs Sso10a1 and Sso10a2 (Figure S4) (Driessen et al., 2016). The winged‐helix domains are identical in structure and they have a C‐terminal alpha helix. Furthermore, Sso10a2 is also a DNA bridger. The Sso10a proteins form dimers with their C‐terminal domain in the form of an anti‐parallel coiled‐coil structure (Chen et al., 2004; Edmondson et al., 2004; Kahsai et al., 2005). On the contrary, the predicted dimer structure of aDCP1/Sul12a is formed by a parallel coiled‐coil structure (Figure 2c). While in Sso10a the DNA‐binding winged‐helix domains end up on the opposite sides of the dimer, in aDCP1/Sul12a they end up together on the same side. The winged‐helix domain of aDCP1/Sul12a is structurally very similar (3.5 Å RMSD) to the winged‐helix domain of the iron‐dependent repressor (IdeR), for which a co‐crystal structure with DNA has been solved (Figure S5) (Marcos‐Torres et al., 2021). Superimposing the co‐crystal structure of IdeR on the dimer prediction of aDCP1/Sul12a shows that the DNA binding sites of aDCP1/Sul12a are located on opposite sides of the dimer, and thus could potentially bind separate DNA duplexes (Figure 2c).

## 5 USING ALPHAFOLD TO HELP CLASSIFY NOVEL NAPS

For many NAPs we do not know how they structure DNA. However, by combining AlphaFold predictions with evolutionary data, we can make predictions as to how these NAPs structure DNA. We will discuss a recently identified and unique NAP, called SymE, and add possible hypotheses as to how it structures DNA.

The majority of NAP research is focused in *E. coli* on the well‐established NAPs, such as the factor for inversion stimulation protein (FIS), HU, and H‐NS. However, new NAPs in *E. coli* are still being identified. One such example is SymE. SymE was originally characterized as a toxin, part of the type I TA *symE*/*symR* toxin‐antitoxin system in *E. coli* (Kawano et al., 2007). SymE is a small protein of 113 amino acids and inhibits cell growth when highly expressed. The reduced cell viability from SymE was originally attributed to its supposed endoribonuclease activity. Its antitoxin, *symR*, encodes a small RNA molecule that inhibits *symE* expression by interacting with the *symE* mRNA, preventing translation by occluding it from the ribosomes. The endoribonuclease activity was a surprising find as the sequence of SymE is similar to the DNA‐binding domain of MazE, the antitoxin of the mazEF toxin‐antitoxin system. Thompson et al. (2022) tried to confirm this endoribonuclease activity by measuring the RNA cleavage levels within *E. coli* when SymE is overexpressed. They found no evidence of endoribonuclease activity. Instead, they discovered that SymE is a DNA‐binding protein. Ectopic overexpression of SymE causes extreme compaction of the nucleoid, similar to H‐NS overexpression. Furthermore, overexpression causes loss of cell viability, likely due to SymE inhibiting DNA replication and transcription. It binds throughout the genome in both inter‐ and intragenic regions and shows no sequence specificity.

No structural data is available on SymE. Based on the AlphaFold predictions, SymE has 3 domains: an N‐terminal disordered tail, a MazE‐like DNA‐binding domain, and a C‐terminal domain consisting of two alpha helices (Figures 2d and S6). SymE dimerizes with its MazE‐like domains into a “saddle” topology similar to MazE (Figure S7) (Zorzini et al., 2015). Interestingly, AlphaFold also confidently predicts a homotetramer structure for SymE, in which both the dimeric MazE‐like domains interact with each other (Figure 2d). In addition, the C‐terminal helices also interact with each other in a “handshake” topology, similar to H‐NS (Qin et al., 2019). This is in agreement with published gel filtration data which suggests that SymE forms tetramers in solution (Thompson et al., 2022). We superimposed the co‐crystal structure of MazE bound to DNA onto the predicted tetramer structure of SymE (Figure 2d). As reference, the MazE “saddle” dimer has two sides: a DNA‐binding side and a non‐binding side. The two MazE‐like dimers of the SymE tetramer interact through their non‐binding side while the DNA binding sides of the “saddle” are pointed away from each other. Consequently, SymE can potentially bind two separate DNA duplexes at each “saddle”, making it a DNA‐bridging NAP. The function of the C‐terminal domains of SymE is unclear. They are strongly positively charged and thus could potentially also bind DNA (Figure S8). Moreover, the solvent‐exposed lysine and arginine residues in the C‐terminal domain are the strongest conserved residues of the domain (Figure S9). Alternatively, they could be domains that facilitate further oligomerization, although AlphaFold does not predict a confident multimer larger than a tetramer. We suspect further multimerization to be possible because the C‐terminal domains could potentially also interact with C‐terminal domains of other SymE tetramers instead of interacting within the tetramer.

## 6 SIMPLE BIOCHEMICAL METHODS FOR CLASSIFICATION

Classifying NAPs into either bending, wrapping, bridging, or nucleofilament formation requires biochemical or microscopy experiments to measure how NAPs structure DNA. A common first assay performed on new NAPs is atomic force microscopy (AFM). AFM is a very powerful assay in which a microscopic needle scans a surface. By measuring the force on the needle we can effectively “see” molecules that are deposited on this surface. As DNA is too small to see with regular light, AFM is often used to see and measure the effects of NAPs on DNA structure. For example, in AFM we see that H‐NS can form bridges between two DNA duplexes (Dame et al., 2000). AFM images of HU show that it can either bend or form nucleoprotein filaments (van Noort et al., 2004). However, AFM is time‐consuming, requires very clean control images of naked DNA, and the images can be difficult to interpret due to possible artifacts, reminiscent of bridged structures, caused by the drying process. We, therefore, recommend a set of cheap and simple in vitro biochemical assays that give unambiguous insights into the architectural properties of NAPs. These assays can be done by any lab as they use commonly available chemicals and enzymes.

One of the oldest assays still commonly used is the electrophoretic mobility shift assay (EMSA). EMSAs are cheap and quick ways to test if your NAP binds DNA. However, EMSAs give little insight into the architectural properties of NAPs. For DNA‐bending NAPs with sequence specificity, the DNA bending angle can be studied by varying the binding site location on the DNA fragment (Thompson & Landy, 1988). Binding sites that are closer to the center of the fragment will yield reduced mobility upon protein binding compared to binding sites at the ends. From this difference in mobility, the DNA bending angle can be calculated. However, these bending angle estimates are not always very precise, as in the case of the catabolite gene activator protein (CAP), because the bending angle of the DNA complex is not directly measured (Kapanidis et al., 2001; Schultz et al., 1991; Thompson & Landy, 1988). Additionally, bending angles for NAPs without a strong sequence preference can not be studied with this assay. Furthermore, EMSAs are not an in‐solution assay as the DNA‐protein complexes are run through a gel, and thus the results of an EMSA are not always representative of how NAPs behave in water. For example, for HU and Cren7 more bound species are observed in EMSAs than can fit on the DNA (Guo et al., 2008; Wojtuszewski et al., 2001). NAPs can also dissociate in the gel, causing smearing. Nonetheless, besides testing general DNA binding, EMSAs can be used to determine the DNA binding site size of a NAP by using small DNA fragments. This can be a time‐consuming task if each fragment is tested individually. We, therefore, recommend using a DNA ladder as a quick and “dirty” way to test multiple DNA fragments at once. The smallest fragment that still shifts away is close in size to the smallest fragment that the NAP can bind. For details on how to perform the EMSA ladder assay, see Hu et al. (2024).

To test if a NAP bends or wraps DNA, a circularization assay can readily be performed. In the circularization assay, the NAP is added to a small (5′ phosphorylated) DNA fragment (<300 bp) and T4 DNA ligase. In the case that the NAP bends or wraps DNA, it will bring the DNA ends close together and a small DNA circle is formed. To distinguish the DNA circles from linear DNA, a T5 exonuclease digestion, resulting in the digestion of linear DNA, is performed, followed by separation through gel electrophoresis. The advantage of a circularization assay is that it requires minimal reagents and is highly sensitive. However, it is purely a qualitative assay and provides no insights into the deeper dynamics of bending. For a quantitative DNA‐bending assay, fluorescence resonance energy transfer (FRET) and luminescence resonance energy transfer (LRET) measurements can be performed (Kapanidis et al., 2001). We want to emphasize that a lack of signal in FRET or LRET assays does not indicate a lack of bending. We therefore recommend performing the circularization assay together with FRET or LRET assays. For details on how to perform circularization assays, see Hodges‐Garcia et al. (1989), Bailey et al. (1999), and Hu et al. (2024).

Currently, no assay exists to readily distinguish between bending or wrapping. An MNase digestion assay can give some limited insights into whether wrapping or bending is occurring, although it is not hard evidence and should always be supported by additional assays. In an MNase assay, a small amount of endo‐exonuclease MNase is added to the NAP and DNA. After a brief incubation, the MNase is inactivated and the DNA is separated through gel electrophoresis. If the NAP protects DNA from MNase digestion, small DNA fragments are visible. These fragments correspond to the DNA‐binding footprint of the NAP. However, not all NAPs protect DNA. It is dependent on how easily the NAP is displaced from the DNA. DNA‐wrapping proteins form a large protein core with an extensive DNA‐binding surface. As a result, they will be more difficult to displace from the DNA than smaller DNA‐binding units with smaller DNA‐binding surfaces. As DNA‐bending proteins only induce small bends into the DNA, they often bind with a small DNA‐binding surface and thus are more readily displaced. Indeed, DNA‐bending NAPs such as HU and Bd0055/HBb give little to no protection in an MNase assay (Hu et al., 2024). In contrast, DNA‐wrapping homologs of HU and Bd0055/HBb, which are HTa and the (hyper)nucleosome histones, respectively, give prominent protected fragments (Grayling et al., 1997; Hocher et al., 2019). For details on how to perform the MNase assay, see Grayling et al. (1997), Hocher et al. (2019), and Hu et al. (2024).

In a bridging assay, the NAP is added to labeled DNA. The most sensitive version of the bridging assay uses radioactively labeled DNA and magnetic‐bead immobilized DNA (van der Valk et al., 2018). The NAP is added to the two labeled DNA substrates, the magnetic beads are pulled down, and radioactivity is measured in the pulled‐down sample. If the NAP bridges DNA, radioactively labeled DNA will be pulled down with the DNA immobilized on the magnetic beads. As this assay involves radioactivity, it is not accessible to most labs today. Simpler, although less sensitive, versions of the bridging assay are easier to perform. You can use a fluorescent label instead of a radioactive label (Shi et al., 2022). Even simpler, you can use just the magnetic‐bead immobilized DNA, or alternatively use cellulose‐immobilized DNA (Zhang et al., 2020). Instead of adding a radioactive or fluorescent label on your second DNA molecule, you use an unlabeled DNA molecule of a different length compared to the magnetic‐bead immobilized DNA. After adding the NAP, the unlabeled DNA, and the magnetic‐bead immobilized DNA together, you pull down the magnetic beads and separate the pulled‐down sample through gel electrophoresis. On gel, you can then see if the unlabeled DNA is present in the pulled‐down sample, which implies DNA bridging by the NAP. For details on how to perform the radioactive‐, fluorescent‐, and cellulose‐immobilized DNA bridging assays, see van der Valk et al. (2018), Shi et al. (2022), and Zhang et al. (2020), respectively.

Finally, we recommend using AlphaFold predictions to gain initial insights into the structure and multimerization of new NAPs. As exemplified by us with SymE (see “Unclassified NAPs”), AlphaFold can give starting hypotheses on how a new NAP might structure DNA. These initial insights give testable hypotheses and thus help speed up experimental work, as shown by Alcorlo et al. (2024) for protein p6. AlphaFold predictions of monomers and multimers can be easily performed with ColabFold (Mirdita et al., 2022): https://github.com/sokrypton/ColabFold.

## 7 DISCUSSION

The prokaryotic genome is compacted by five possible mechanisms: supercoiling, phase separation, charge neutralization, macromolecular crowding, and NAPs (Joyeux, 2015). Of these five mechanisms, NAPs are special as they can give rise to the hierarchical genome organization as observed in *E. coli*. However, the contribution of specific NAPs to this genome organization is often unclear. This is due to the high amount of redundancy of NAPs within cells. Cells encode a large number of NAPs with overlapping architectural roles: NAPs that bend DNA, wrap DNA, bridge multiple DNA duplexes, and form nucleoprotein filaments. Most studies try to identify the in vivo role of NAPs by studying the changes in genome structure after deletion of the NAP. The effects are often subtle and/or difficult to interpret because the loss of one NAP is compensated in the cell by other NAPs with similar architectural functions. The high amount of NAP redundancy is probably the biggest obstacle that limits our understanding of how the genome gets structured in vivo by NAPs.

In contrast, in vitro studies of NAPs give clearer insights into their possible architectural properties. Common in vitro methods to identify the architectural properties are crystallography, EMSAs, and AFM. We like to emphasize that these methods do not necessarily represent how NAPs behave in their native state: in‐solution (water). Instead, we recommend the use of more in‐solution methods to unambiguously characterize the in vitro properties of NAPs, which also helps to identify possible artifacts in crystallography or AFM data. This is especially relevant now as an increasing number of new NAPs are being identified from the rapidly growing amount of metagenomic data, new NAPs which can not be studied or are difficult to study in vivo. However, in vitro data is only one step in understanding the function of NAPs in genome organization. While in vitro data helps to categorize NAPs based on their architectural properties, the in vitro data is difficult to translate to in vivo behavior of NAPs. This is complicated by the fact that the contribution of specific NAPs to genome organization is likely dependent on other NAPs that are present in the cell, combinatory effects which are not studied in vitro. How we should combine in vitro and in vivo studies has been discussed for decades now with no clear solutions available today. New techniques, such as single particle cryo‐electron microscopy (cryo‐EM) and cryogenic electron tomography (cryo‐ET) look promising as they allow us to study the in vitro structures of single NAP molecules and the in vivo structures that these NAPs form with the genome (Cheng, 2018; Liedtke et al., 2022). While the prospect of crossing the in vitro‐in vivo gap still seems far away, currently, in vitro studies give us a well‐defined foothold from which we could try to understand how NAPs organize the genome.

## AUTHOR CONTRIBUTIONS

**Samuel Schwab:** Conceptualization; writing – original draft; writing – review and editing; investigation; formal analysis; visualization. **Remus T. Dame:** Conceptualization; supervision; writing – review and editing; funding acquisition.

## CONFLICT OF INTEREST STATEMENT

The authors declare no conflict of interest.

## ETHICS STATEMENT

All authors agree with the contents of this article.

## Supporting information


Data S1.

## Data Availability

The data that support the findings of this study are openly available in 4TU at https://doi.org/10.4121/86b33bd9‐d717‐4890‐973f‐06d22b6e8b11.
