# Supplementary material for: Identification, characterization and classification of prokaryotic nucleoid‐associated proteins
Source: Mol Microbiol. 2024 Jul 22;123(3):206–17. doi: 10.1111/mmi.15298 (PMC11894785; doi:10.1111/mmi.15298)
Supplement: Supplementary file 1 — Data S1. [file MMI-123-206-s001.pdf]

Identification, characterization and classification of prokaryotic Nucleoid-Associated Proteins

Running title: To be or not to be a NAP

Samuel Schwab[1], Remus T. Dame[1,*]

[1]Leiden Institute of Chemistry, Leiden University, Einsteinweg 55, 2333CC Leiden, The Netherlands; Centre for Microbial Cell Biology, Leiden University, Leiden, The Netherlands; Centre for Interdisciplinary Genome Research, Leiden University, Leiden, The Netherlands

Supplementary Information

*Corresponding author: rtdame@chem.leidenuniv.nl

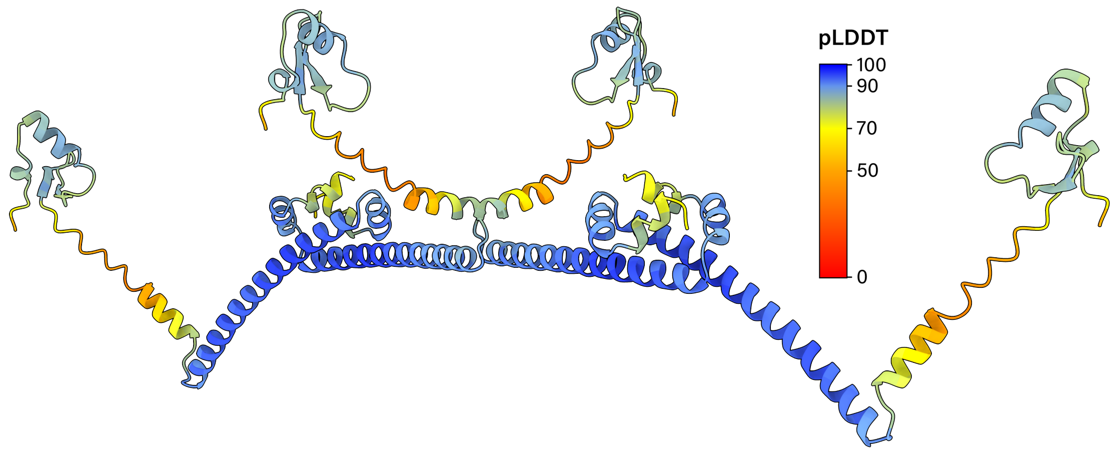

(a)

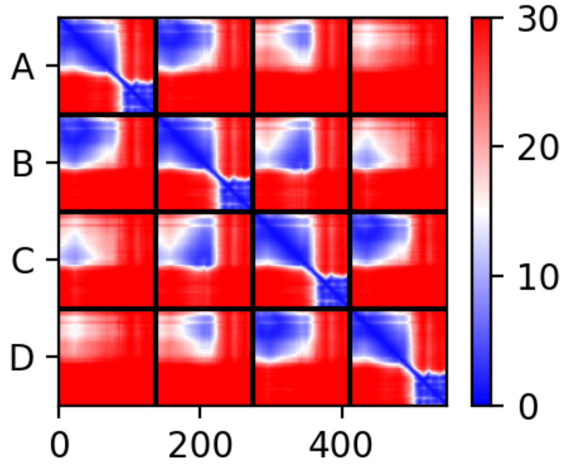

(b)

Figure 1: (a) The homotetramer of H-NS from *Escherichia coli* as predicted by AlphaFold2. Each residue is colored by its predicted local distance difference test (pLDDT) value. (b) Predicted aligned error plot for the homotetramer prediction of H-NS. The value at (x,y) is the expected distance error (Å) of residue x relative to residue y when residue y is aligned to the true structure.

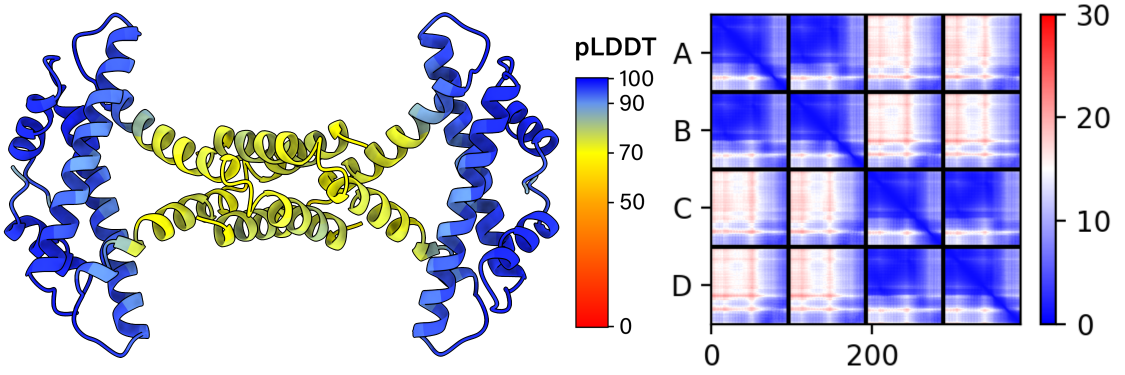

(a) (b)

Figure 2: (a) The homotetramer of MJ1647 from *Methanocaldococcus jannaschii* as predicted by AlphaFold2. Each residue is colored by its predicted local distance difference test (pLDDT) value. (b) Predicted aligned error plot for the homotetramer prediction of MJ1647. The value at (x,y) is the expected distance error (Å) of residue x relative to residue y when residue y is aligned to the true structure.

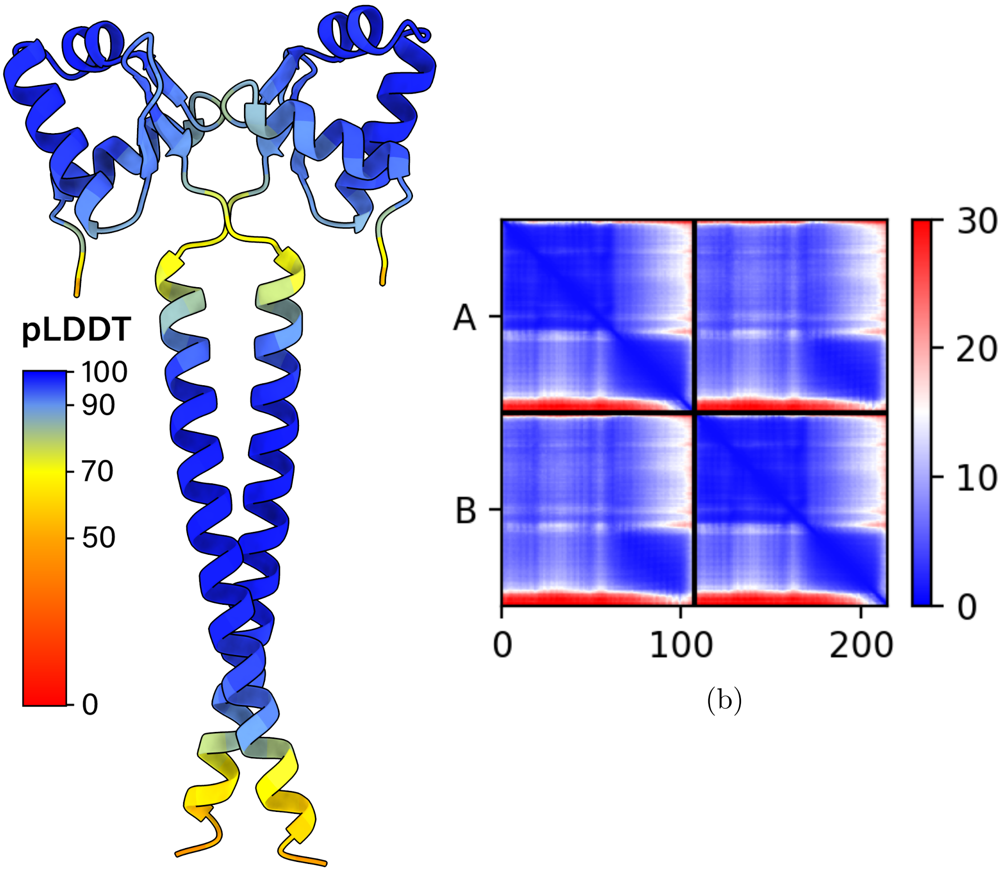

(a)

(b)

Figure 3: (a) The homodimer of Sul12a from *Sulfolobus acidocaldarius* as predicted by AlphaFold2. Each residue is colored by its predicted local distance difference test (pLDDT) value. (b) Predicted aligned error plot for the homodimer prediction of Sul12a. The value at (x,y) is the expected distance error (Å) of residue x relative to residue y when residue y is aligned to the true structure.

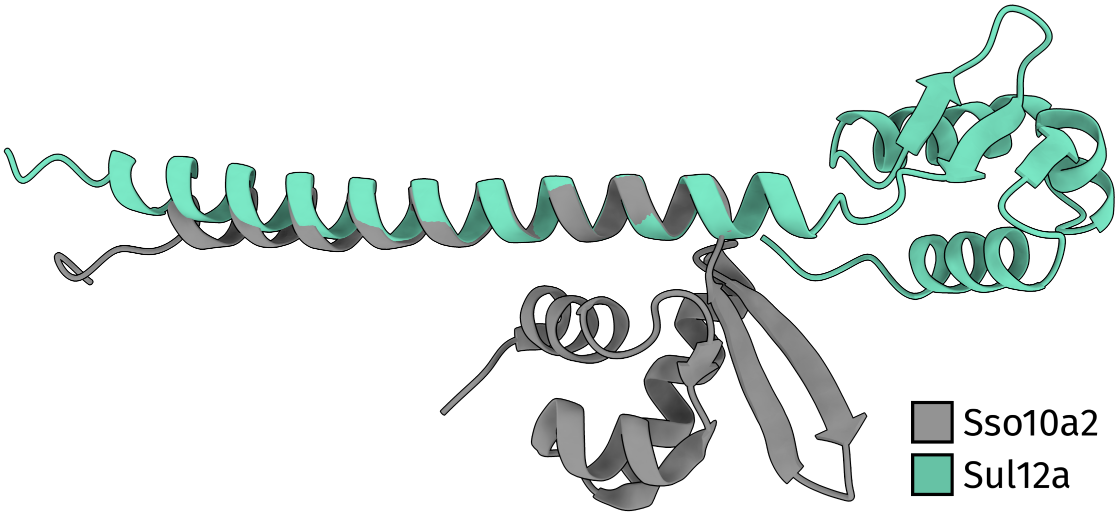

Figure 4: Superimposed structures of Sso10a2 (PDB: 4HW0) (Driessen et al., 2016) and Sul12a as predicted by AlphaFold in grey and green respectively.

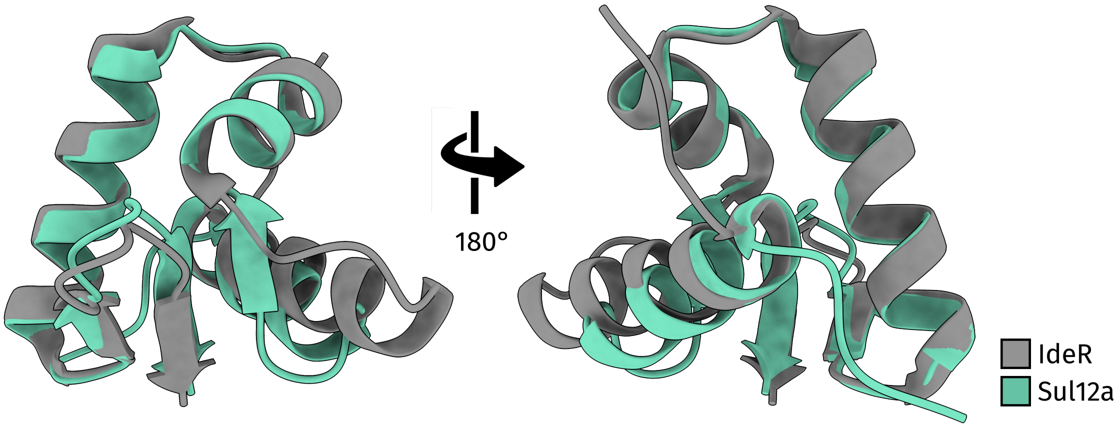

Figure 5: Superimposed structures of the winged-helix domains of IdeR (PDB: 7B1Y) (Marcos-Torres et al., 2021) and Sul12a as predicted by AlphaFold in grey and green respectively.

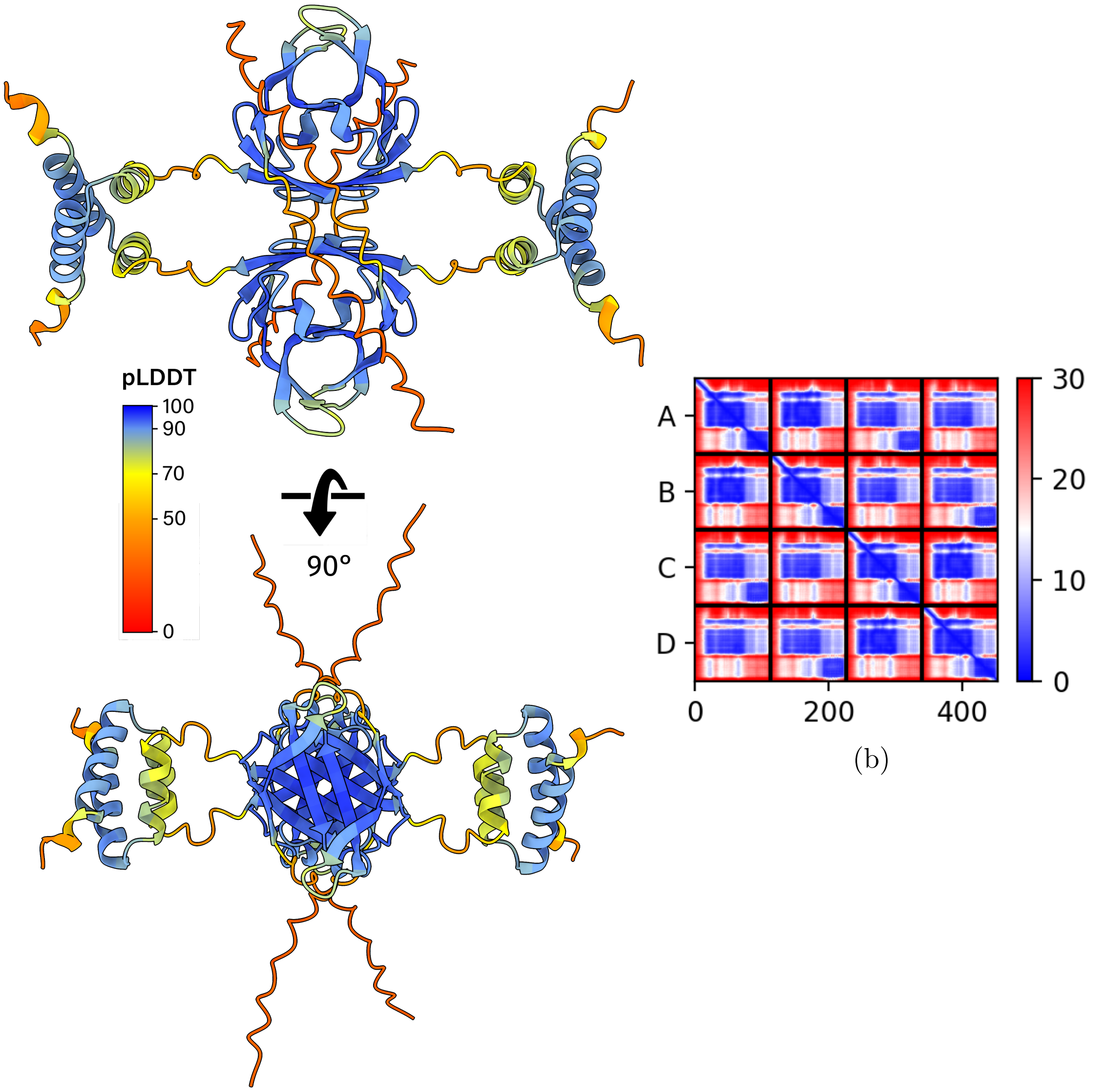

(b)

(a)

Figure 6: (a) The homotetramer of SymE from *Escherichia coli* as predicted by AlphaFold2. Each residue is colored by its predicted local distance difference test (pLDDT) value. Values below 50 are interpreted as a prediction of disorder. (b) Predicted aligned error plot for the homotetramer prediction of SymE. The value at (x,y) is the expected distance error (Å) of residue x relative to residue y when residue y is aligned to the true structure.

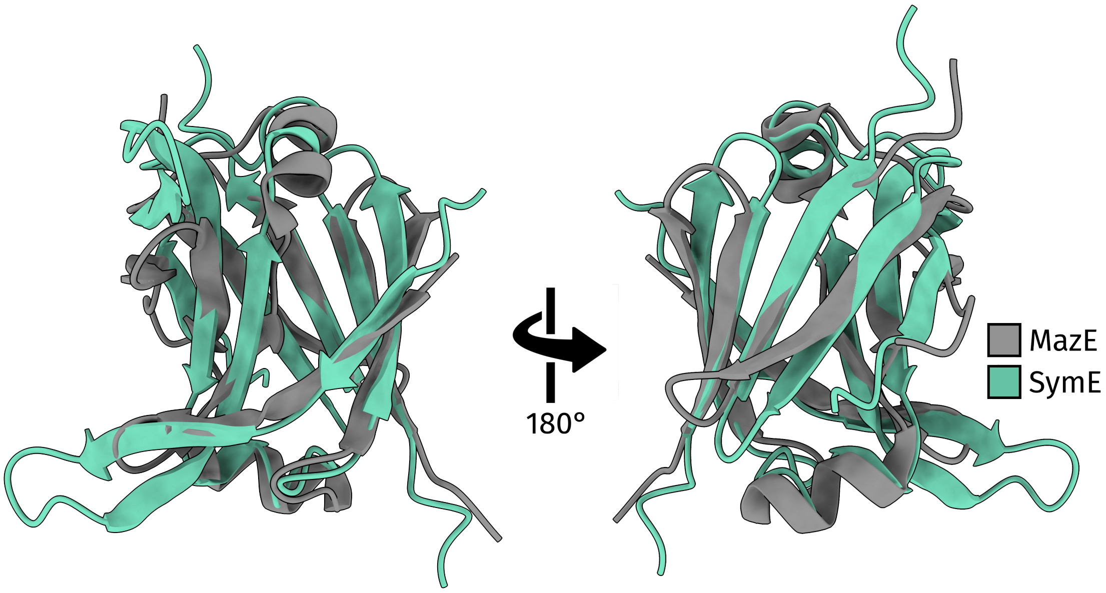

Figure 7: Superimposed structures of the MazE (PDB: 2MRU) (Zorzini et al., 2015) and the MazE-like domain of SymE as predicted by AlphaFold in grey and green respectively.

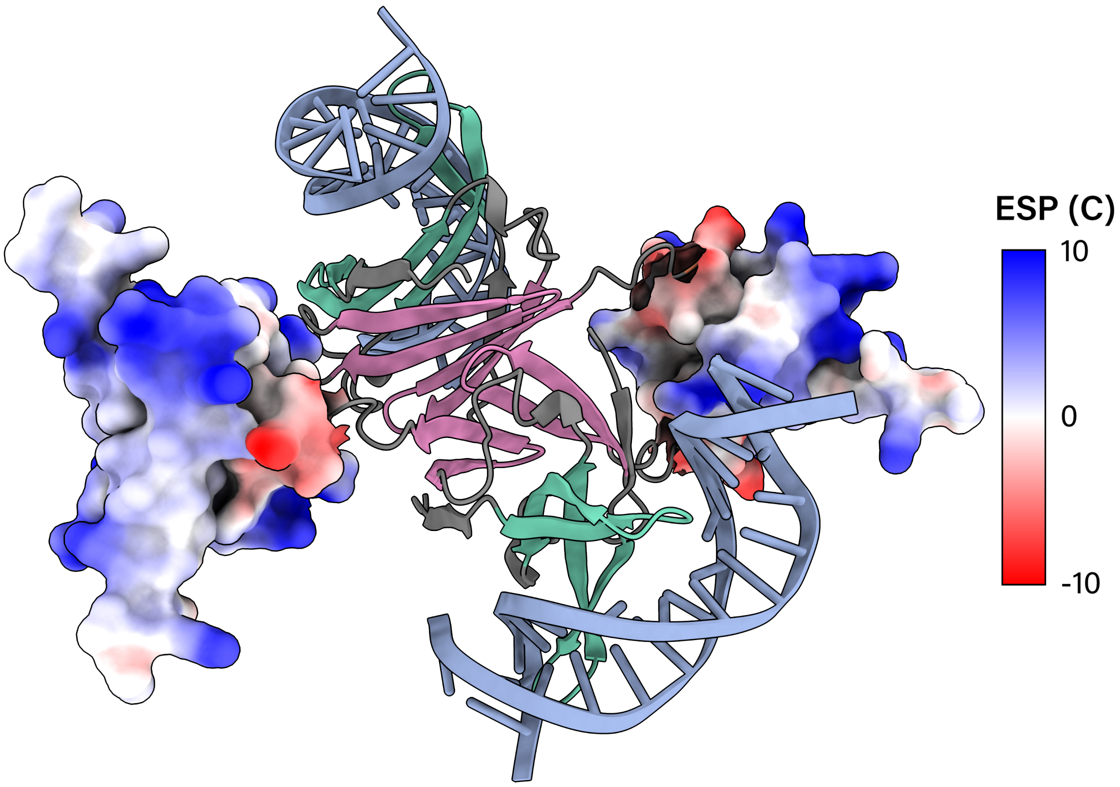

Figure 8: The homotetramer of SymE from *Escherichia coli* as predicted by AlphaFold2. The (disordered) N-terminal tail of SymE is hidden to reduce visual clutter. The DNA, the DNA binding domain, and the dimer-dimer domain are colored blue, green, and pink respectively. The C-terminal domains are colored by Coulombic electrostatic potential (ESP).

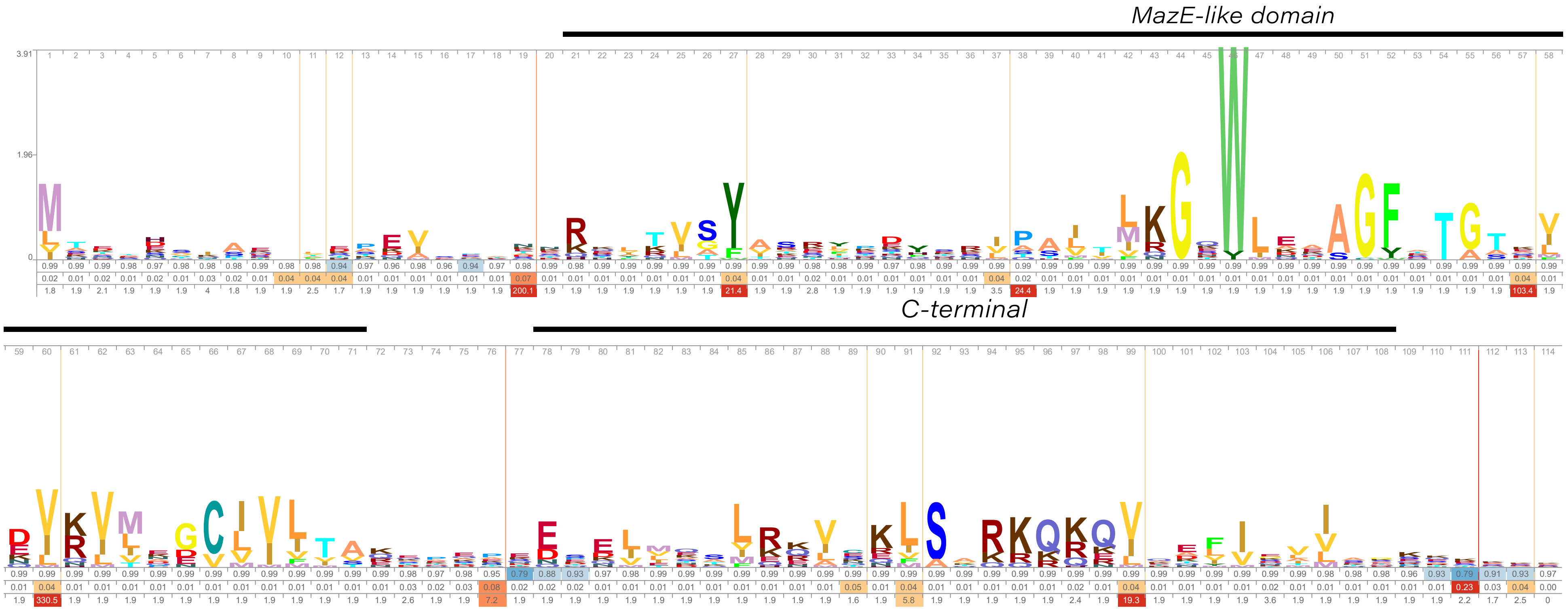

Figure 9: Logo representation of the SymE HMM profile. Only residues with scores above background frequency are shown. The occupancy probability, insert probability, and insert length values are below the residues. The domains are labeled above the residues.

Supplementary Material and Methods

AlphaFold predictions

All predictions were performed with LocalColabFold using the v3 AlphaFold2-Multimer model weights on the high-performance computing facility ALICE at Leiden University (Mirdita et al., 2022; Jumper et al., 2021; Evans et al., 2021). Multiple sequence alignments (MSAs) were generated on the MMseqs2 server provided by ColabFold (Mirdita et al., 2022). No templates were used in the predictions. Predictions were done with 6 recycles for SymE, 12 recycles for MJ1647 and Sul12a, and 30 recycles for H-NS. The predicted structures were not relaxed by AlphaFold's AMBER forcefield.

Hidden Markov Profiles (HMM)

SymE homologs were collected using EMBL-EBI's online jackhmmer server with default settings: `https://www.ebi.ac.uk/Tools/hmmer/search/jackhmmer`. SymE was used as the starting query. One iteration of jackhmmer was run. All SymE homologs were aligned in a multiple sequence alignment using Muscle v5 with the Super5 algorithm (Edgar, 2022). From the multiple sequence alignment, an HMM profile was generated with HMMER (v3.3.2) (Eddy, 2011). With Skylign a logo was generated from the HMM profile (Wheeler et al., 2014).

Data availability

The predicted AlphaFold structures shown in this article and the HMM profile are available from the 4TU repository (`https://data.4tu.nl`) with DOI 10.4121/86b33bd9-d717-4890-973f-06d22b6e8b11.

References

Driessen, R.P.C., Lin, S.N., Waterreus, W.J., van der Meulen, A.L.H., van der Valk, R.A., Laurens, N., Moolenaar, G.F., Pannu, N.S., Wuite, G.J.L., Goosen, N., and Dame, R.T. Diverse architectural properties of Sso10a proteins: Evidence for a role in chromatin compaction and organization. *Scientific Reports*, 6(1):29422, July 2016. ISSN 2045-2322. doi: 10.1038/srep29422. URL `https://www.nature.com/articles/srep29422`.

Eddy, S.R. Accelerated profile HMM searches. *PLoS Computational Biology*, 7 (10), 2011. ISSN 1553734X. doi: 10.1371/journal.pcbi.1002195.

Edgar, R.C. Muscle5: High-accuracy alignment ensembles enable unbiased assessments of sequence homology and phylogeny. *Nature Communications*, 13(1): 1–9, 2022. ISSN 20411723. doi: 10.1038/s41467-022-34630-w.

Evans, R., O'Neill, M., Pritzel, A., Antropova, N., Senior, A., Green, T., Žídek, A., Bates, R., Blackwell, S., Yim, J., Ronneberger, O., Bodenstein, S., Zielinski, M., Bridgland, A., Potapenko, A., Cowie, A., Tunyasuvunakool, K., Jain, R., Clancy, E., Kohli, P., Jumper, J., and Hassabis, D. Protein complex prediction with alphafold-multimer. *bioRxiv*, 2021. doi: 10.1101/2021.10.04.463034. URL `https://www.biorxiv.org/content/early/2021/10/04/2021.10.04.463034`.

Jumper, J., Evans, R., Pritzel, A., Green, T., Figurnov, M., Ronneberger, O., Tunyasuvunakool, K., Bates, R., Žídek, A., Potapenko, A., Bridgland, A., Meyer, C., Kohl, S.A., Ballard, A.J., Cowie, A., Romera-Paredes, B., Nikolov, S., Jain, R., Adler, J., Back, T., Petersen, S., Reiman, D., Clancy, E., Zielinski, M., Steinegger, M., Pacholska, M., Berghammer, T., Bodenstein, S., Silver, D., Vinyals, O., Senior, A.W., Kavukcuoglu, K., Kohli, P., and Hassabis, D. Highly accurate protein structure prediction with AlphaFold. *Nature*, 596 (7873):583–589, 2021. ISSN 14764687. doi: 10.1038/s41586-021-03819-2. URL `http://dx.doi.org/10.1038/s41586-021-03819-2`.

Marcos-Torres, F.J., Maurer, D., Juniar, L., and Griese, J.J. The bacterial iron sensor IdeR recognizes its DNA targets by indirect readout. *Nucleic Acids Research*, 49(17):10120–10135, September 2021. ISSN 0305-1048. doi: 10.1093/nar/gkab711. URL `https://doi.org/10.1093/nar/gkab711`.

Mirdita, M., Schütze, K., Moriwaki, Y., Heo, L., Ovchinnikov, S., and Steinegger, M. ColabFold: making protein folding accessible to all. *Nature Methods*, 19(6): 679–682, June 2022. ISSN 1548-7105. doi: 10.1038/s41592-022-01488-1. URL `https://www.nature.com/articles/s41592-022-01488-1`.

Wheeler, T.J., Clements, J., and Finn, R.D. Skylign: A tool for creating informative, interactive logos representing sequence alignments and profile hidden Markov models. *BMC Bioinformatics*, 15(1):1–9, 2014. ISSN 14712105. doi: 10.1186/1471-2105-15-7.

Zorzini, V., Buts, L., Schrank, E., Sterckx, Y.G., Respondek, M., Engelberg-Kulka, H., Loris, R., Zangger, K., and van Nuland, N.A. Escherichia coli antitoxin MazE as transcription factor: insights into MazE-DNA binding. *Nucleic Acids Research*, 43(2):1241–1256, January 2015. ISSN 0305-1048. doi: 10.1093/nar/gku1352. URL `https://doi.org/10.1093/nar/gku1352`.
